# Sclerema Neonatorum in a Term Infant: A Case Report and Literature Review

**DOI:** 10.1155/2020/8837064

**Published:** 2020-12-30

**Authors:** Gloria Nakalema, Walufu Ivan Egesa, Patrick Kumbowi Kumbakulu, Martin Nduwimana, Amnia Diaz Anaya, Mirembe Stephen Kizito, Daniel Kavuma

**Affiliations:** ^1^Department of Paediatrics and Child Health, Faculty of Clinical Medicine and Dentistry, Kampala International University, Kampala, Uganda; ^2^Department of Pathology, Faculty of Clinical Medicine and Dentistry, Kampala International University, Kampala, Uganda; ^3^Department of Dermatology, Faculty of Clinical Medicine and Dentistry, Kampala International University, Kampala, Uganda; ^4^Department of Surgery, Faculty of Clinical Medicine and Dentistry, Kampala International University, Kampala, Uganda

## Abstract

Sclerema neonatorum (SN) is a rare form of panniculitides with an obscure incidence, aetiology, and pathogenesis. This condition is peculiar to preterm neonates, especially those with comorbidities such as sepsis, congenital anomalies, congenital heart disease, and gastrointestinal and respiratory diseases. Usually, it presents within the first seven days of life, but may develop a few weeks later. Typically, SN manifests with diffuse hardening of the skin and subcutaneous tissues that subsequently bind to the underlying muscle and bone, often beginning in the buttocks, thighs, or trunk, and progressing to other body parts, but sparing the soles, palms, and genitalia. Diagnosis is based on clinical characteristics. It has been associated with a high mortality, despite aggressive use of various treatment modalities such as antibiotics, steroids, fresh blood transfusion, exchange blood transfusion, and intravenous immunoglobins. This is a report of a macrosomic term neonate who presented with typical clinical and histopathological features of SN.

## 1. Introduction

Sclerema neonatorum (SN) is an uncommon form of panniculitides with an invariably high fatality [1]. SN predominantly affects preterm neonates and characteristically presents with diffuse nonedematous hardening of the skin and subcutaneous tissues [2, 3]. The first description of this condition was made by Usembenzius in 1718, who coined the term ‘acute sclerema' [4]. It was Underwood who later gave a more classic description in 1784, terming it ‘skinbound' [5]. For more than 100 years, confusion has persisted among researchers regarding SN, subcutaneous fat necrosis of the newborn (SCFN), and scleredema, terms that have been used since the 19th century [6, 7]. The exact incidence, aetiology, and pathogenesis of SN remain unknown. Nonetheless, researchers have proposed controversial theories that SN may occur as a result of increase in saturated fat, defective fat metabolism, inherent abnormality in adipocytes or connective tissue, or a sign of disease toxicity [7].

This is a report of a macrosomic term neonate with a cleft palate, sepsis, and meconium aspiration syndrome who developed SN.

## 2. Case Description

We report the case of a male infant delivered by emergency caesarean section under spinal anaesthesia, due to foetal distress at 40 weeks of gestation. He was born to a 30-year-old HIV-positive but nondiabetic G4 + P3 + 0 mother on highly-active antiretroviral therapy (tenofovir, lamivudine, and efavirenz) who attended four antenatal-care visits and had an uneventful pregnancy. The mother did not have syphilis and reported no familial diseases. However, she ingested unspecified herbal medicines during labour, and foul-smelling meconium was observed during delivery, although she had no recent abnormal vaginal discharge. The infant weighed 5.5 kg at birth, and had an Apgar score of 5 at 1 minute and 7 at 5 minutes. Successful resuscitation was performed using gentle airway suctioning and bag-and-mask positive pressure ventilation.

**Figure 1:**
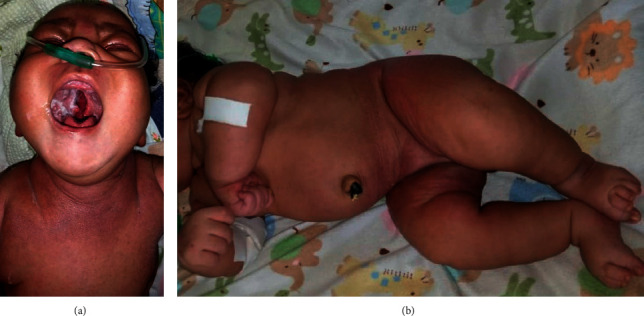
Photographs of the neonate with a cleft palate and diffusely indurated purplish skin in the neck region (a), thighs, buttocks, and groin (b) that could not be pinched or pitted.

At 30 minutes of life, the infant was admitted to the neonatal intensive care unit at Kampala International University Teaching Hospital due to severe respiratory distress. The initial physical examination revealed severe respiratory distress, meconium-stained skin, left cleft palate (Figure 1(a)), and bilaterally undescended testis which were also not palpable in the inguinal canals. The patient was normothermic, had no dysmorphic features, and cardiac auscultation was normal. On day 2 of life, diffuse hardening of the skin was noticed, initially involving the lower back, with gradual progression to the trunk and extremities within 48 hours of onset. However, the skin of the palms, soles, face, and genitalia was spared. Affected skin was smooth, purplish (Figures 1(a) and 1(b)), indurate, and nonpitting and could not be picked up and pinched. The infant also had limited movements of the extremities and had no convulsions observed, and the anterior fontanelle was flat.

**Figure 2:**
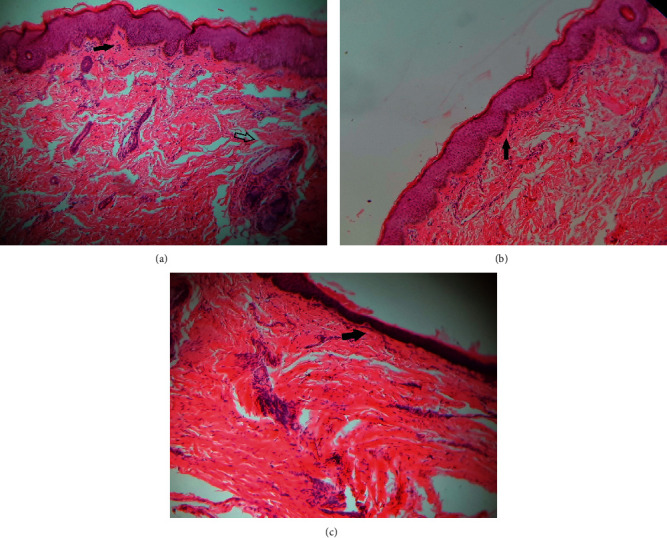
Histology findings of skin biopsy showing extensive fibrosis with dense subepithelial collagen deposition, sparse inflammatory cell infiltration (black arrow), and entrapment of the dermal adnexa between the collagen fibers (white arrow) (a, b). Atrophy of the epidermis and loss of epidermal papilla (c) (H&E stain ×10).

During the period of residence, a diagnosis of a macrosomic infant with meconium aspiration syndrome, early-onset neonatal sepsis, and SN was made. The patient received oxygen therapy, nevirapine prophylaxis, intravenous cefotaxime, intravenous ampicillin, and intravenous fluids. The infant later suffered from infected wounds at cannulation sites, for which he was given cloxacillin, and then improved. The total leucocyte count was 71,000/*μ*L, hemoglobin was 171 g/L, and platelet count was 212,000/*μ*L on the first day of life, whereas the serum glucose level ranged from 2.5 mmol/L to 13.6 mmol/L throughout the course of hospitalisation. C-reactive protein (CRP) level, serum electrolytes, and blood cultures were not performed. The chest X-ray was normal. A punch biopsy specimen of affected skin and subcutaneous tissue on the trunk was taken, and subsequent histology revealed mild infiltration of lymphocytes, severe deposition of collagen in the dermis, entrapment of the dermal adnexa between the collagen fibers, and atrophy of the epidermis and glands. These findings suggested sclerema (Figure 2).

The infant was discharged on the 36th day of life after respiratory symptoms had resolved and was breastfeeding normally. On the other hand, regression of skin hardening had not yet been observed. Unfortunately, the infant died at home aged 7 weeks of life, following a cultural practice of ‘false teeth' extraction, also referred to as “ebiino.” This procedure is frequently performed by traditional healers, who use nonsterile sharp objects to manipulate the canine tooth buds.

## 3. Discussion

Sclerema neonatorum is a rare and severe skin condition that predominantly affects preterm neonates, although case reports of term neonates have been published [3, 8, 9]. Often, the condition occurs in ill neonates with comorbidities such as congenital anomalies, sepsis, congenital heart disease, and respiratory or gastrointestinal diseases [2, 3, 9–11]. A relationship between HIV exposure, highly-active antiretroviral therapy (HAART), and SN has not been reported. In this case, the neonate was born at term and had a congenital anomaly and sepsis.

The diagnosis of SN is generally clinical, and signs usually develop during the first 7 days of life, although this may occur a few weeks to months later [3, 8, 10–12]. Sclerema neonatorum is typically characterized by hardening of the skin and subcutaneous tissues that subsequently bind to the underlying muscle and bone, compromising respiration and feeding and leading to death in the majority of cases [2, 6, 7]. This process occurs where fat is plentiful [13] and typically begins in the buttocks, thighs, or trunk but may rapidly progress to involve other body parts, sparing the soles, palms, and genitalia [3]. The skin of affected neonates is usually nonedematous, smooth, cool, tense, mottled, purplish, and hard [6]. Because the skin is bound to subjacent subcutaneous tissue, including muscle and bone, it cannot be pitted by pressure or pinched into a fold [2, 7], and patients may develop stiff limbs [6]. The neonate discussed herein developed characteristic features of SN including woody-hard skin that could not be pitted or pinched and reduced mobility of extremity joints. His palms, soles, and genital area were unaffected, which suggested SN. A PubMed® search was performed for similar cases of SN that were published in the English language literature since 1995 (Table 1).

Definitive diagnosis of SN requires a histopathological study. Histology evaluation of skin biopsy shows thickening of the trabeculae supporting the subcutaneous adipose tissue and a sparse infiltration of lymphocytes, histiocytes, and multinucleate giant cells, which is attributable to a poor immunological response [7, 14]. In addition, subcutaneous fibrosis, thinning of the epidermis, atrophy of the rete pegs, dense deposition of collagen in the dermis [15], and fine, needle-shaped crystals may be observed in adipocytes [13, 16, 17]. The constellation of clinical and histopathologic features described above provided the basis for a diagnosis of SN in our case.

The most important differential diagnosis of SN is SCFN, a self-limiting disorder that is characterised by isolated or multiple, firm, inflamed, skin-coloured to purple subcutaneous nodules, most commonly involving the back, buttocks, and extremities [18]. The lesions move freely, are not attached to muscles and bone, and generally do not spread beyond the initial areas of involvement [19]. SCFN usually occurs within the first 7 days in term and postterm infants [14, 18, 20]. SN should also be distinguished from scleredema, a self-limiting condition with an onset during the first week of life, invariably affecting preterm neonates. It manifests with generalized, firm, pitting edema with an increase in the volume of affected parts. Skin biopsy reveals inflammatory infiltrate of lymphocytes and histiocytes with marked edema of skin and subcutaneous tissues [7].

Management of SN requires intensive efforts to correct fluid imbalance and dyselectrolytemia, ventilatory support, temperature control, and psychosocial support [7, 12, 21]. To date, there are no defined treatment guidelines for SN, despite documentation of the beneficial effects of treatment modalities such as parenteral antibiotics, steroids [6, 22, 23], fresh blood transfusion [3], exchange blood transfusion [9, 24, 25], and immunoglobins [12]. This may be explained by the lack of sufficiently plausible evidence from randomized-controlled trials. Therefore, management is largely supportive, directed to the underlying cause [26]. The neonate we present was conservatively treated with intravenous fluids and antibiotics for presumed sepsis. This approach is similar to that reported by Battin et al. [27], Park and Kim [8], and Loberger et al. [26], who presented case reports of term neonates that survived after a conservative approach. In contrast to the latter infants who survived, the death of the infant we have presented was attributed to a dangerous traditional practice. Therefore, larger studies are needed to standardize therapy for SN.

The prognosis of neonates with SN is universally poor, with a case fatality ranging from 82% to 98% [11, 28], attributable to the underlying disease and restriction of respiration due to skin tightness [7]. Long-term integumentary complications have not been reported among survivors [26].

## 4. Conclusion

SN is an extremely rare and poorly understood complication of critically-ill infants in the age of advanced neonatal care. While SN primarily affects preterm infants, especially those with comorbidities, it may also affect term infants. As in the current case, SN should be suspected in neonates who manifest with diffuse, waxy, nonpitting hardening of the skin that spares the palms, soles, face, and genitalia. Our patient was conservatively treated with antibiotics, without the use of systemic steroids, exchange transfusion, or intravenous immunoglobins, but later died following a high-risk traditional procedure.

## Figures and Tables

**Table 1:** Treatment and outcomes of sclerema neonatorum over the last 25 years.

No	Author(s) and year	Number of patients	Gestation	Management	Outcome
1	Sadana et al., 1997	20		Exchange transfusion with fresh blood^a^	50% mortality
2	Battin et al., 2002	1	Term	Conservative	Survived
3	Zeb et al., 2009	51	Preterm	Emollients^a^	98% mortality
4	Buster et al., 2013	1	Term	IVIG^a^	Died
5	Park and Kim, 2017	1	Term	Conservative, moisturizer	Survived; mild improvement by 12 months
6	Spohn et al., 2016	1	Preterm	Conservative^a^	Died
7	Shrestha et al., 2017	1	Preterm	Intravenous steroids^a^	Survived
8	Loberger et al., 2019	1	Term	Conservative^a^	Survived
9	Younes et al., 2019	1	Preterm	Steroids^a^	Not documented

^a^Antibiotics given to neonates with sepsis. IVIG, intravenous immunoglobin.

## Data Availability

The data used to support the findings of this study are available from the corresponding author upon request.
